# Factors associated with dengue fever outbreak in Dire Dawa administration city, October, 2015, Ethiopia - case control study

**DOI:** 10.1186/s12889-019-7015-7

**Published:** 2019-05-28

**Authors:** Luna Habtamu Degife, Yoseph Worku, Desalegn Belay, Abyot Bekele, Zegeye Hailemariam

**Affiliations:** 1 Center for Public Health Emergency Management, Ethiopian Public Health Institute, PO.BOX: 1242, Addis Ababa, Ethiopia; 2 Saint Paul’s Hospital Millennium Medical College, Addis Ababa, Ethiopia; 3 Ethiopian Field Epidemiology and Laboratory Training Program, Addis Ababa, Ethiopia

**Keywords:** Dengue fever, Dire Dawa, Outbreak, Risk factors

## Abstract

**Background:**

Dengue Fever (DF) is underrecognized mosquito borne viral disease prevalent in tropical and subtropical regions. In 2013, Ethiopia reported the first confirmed DF outbreak in Dire Dawa city which affected 11,409 people. During the outbreak investigation, we determined factors associated with DF and implemented control measures.

**Methods:**

We conducted a 1:2 un-matched case control study from October 7–15/2015. Case was any person with fever of 2–7 days and more than two symptoms: headache, arthralgia, myalgia, rash, or bleeding from any part of the body. We recruited participants using purposive sampling from health facilities and used structured questionnaire to collect data. Multiple logistic regression analysis was conducted to control confounders and to identify factors associated with DF. Sixty-nine serum-samples were tested by Enzyme-Linked Immunosorbent Assay (ELISA).

**Results:**

We enrolled 210 participants (70 cases and 140 controls) in the study. Females accounted for 51.4% of cases and 57.1% of controls. The mean age was 23.7 ± 9.5 standard deviation (SD) for cases and 31.2 ± 13 SD for controls. Close contact with DF patient (Adjusted odds ratio [AOR] =5.36, 95% confidence interval [CI]: 2.75–10.44), nonuse of bed-nets (AOR = 2.74, 95% CI: 1.06–7.08) and stagnant water around the village (AOR = 3.61, 95% CI: 1.31–9.93) were independent risk factors. From the samples tested, 42 were confirmed positive.

**Conclusions:**

Individuals who live with DF patient, around stagnant water and do not use bed nets are at high risk of contracting the disease. Health education on DF prevention was given and mosquito breeding sites were drained. Strong vector prevention strategies are recommended by enhancing the existing malaria prevention and control program.

**Electronic supplementary material:**

The online version of this article (10.1186/s12889-019-7015-7) contains supplementary material, which is available to authorized users.

## Background

Classic dengue fever (DF), or “break bone fever,” is the most rapidly spreading arboviral disease with the highest prevalence in the tropical and subtropical regions of the world [1]. The virus belongs to the genus Flavivirus, family Flaviviridae with 4 serotypes known as dengue virus type 1, 2, 3 and 4 [2]. DF including the severe forms, dengue hemorrhagic fever (DHF) and dengue shock syndrome (DSS) can be caused by any of the serotypes. A person infected with one serotype is likely to develop DHF if later on infected with a different serotype but protected for the similar serotype [3]. It is characterized by acute onset of high fever 3–14 days after the bite of an infected female *Aedes aegypti*, a domestic day-biting mosquito. Even though most Dengue infections are asymptomatic or cause a relatively mild disease, some DF infections may result in severe and potentially life-threatening disease [4]. With early diagnosis and proper management, the case-fatality rate (CFR) of DHF is generally under 1%, but the CFR may be over 10% once shock develops [5].

More than 50% of the world’s population are at risk and over 125 countries are endemic to DF. The global incidence of DF has increased and expanded geographically by 30 folds from the past 50 years. Worldwide, it is estimated that 50 to 270 million dengue infections occur every year, out of which two million cases are severe DHF and 21,000 result in death [6, 7]. Determinant factors of dengue global epidemiology trends include, but are not limited to: Demographic changes, increased urban population, modern transportation and changes in public health policies [6].

In Africa, laboratory diagnostics is not done for most of the febrile illnesses and is taken as malaria. Even though outbreaks of dengue have been reported, there is limited data on the incidence and prevalence of the disease. The first report of Dengue in Africa was in the late 19th and early 20th centuries from Burkina Faso, Zanzibar, Senegal, Egypt and South Africa [8].

Despite the little documented data in Africa, the disease is endemic to 34 countries with all four dengue virus serotypes in circulation. Available data suggest that *Aedes aegypti* mosquitoes are known to be present in all but five countries [9]. In Ethiopia, DF was diagnosed only among travelers returning to countries to which dengue was not endemic but never reported as an outbreak locally [8] till September 2013, when the first major outbreak occurred in Diredawa administration city with a total of 11,409 cases [10]. Therefore, the aim of this study is to determine factors associated with DF outbreak in Diredawa administration city in 2015.

## Objective

To determine the risk factors associated with Dengue Fever in Diredawa administration city, October 2015.

## Methods

Dire Dawa administration city is found 515 Kilometers south east of Addis Ababa, the capital of Ethiopia. The council has a total approximate population of 400,000 and nine operational districts (4 rural and 5 urban districts). It is further classified into 10 urban and 37 rural kebeles, the smallest administrative unit in Ethiopia. About 70% of the people live in the city while the rest are in the surrounding rural kebeles. Moreover, the council has 2 government owned Hospitals, 15 health centers and 31 health posts. Additionally, there are 3 private hospitals and 21 private clinics. There are both arid and semiarid climatic zones in the council. The north eastern part of the city is relatively sparsely populated lowland exhibiting agro-pastoral and pastoral system, and the southeastern part uses mixed farming system. The city lies between 1000 to 2000 m above sea level with an average monthly temperature of 24.8 ^o^C and annual rainfall of 604 mm [10, 11]. The warm climate of the City is favorable for the transmission of vector borne diseases like malaria and DF.

An unmatched case-control study design was used to investigate the outbreak. All residents of Diredawa Administration city were the source population. Cases were either confirmed or epidemiologically linked Dengue Fever cases while Controls were all people without Suspected DF symptoms. Additionally, confirmed cases were suspected cases with laboratory confirmation (positive IgM antibody, rise in IgG antibody titers, positive PCR or viral isolation). Moreover, epidemiologically linked cases were suspected cases presented at the location of ongoing outbreak within previous two weeks of onset of an acute febrile illness or dengue, or association in time and place (e.g., household member, family member, classmate, or neighbor) with a confirmed or probable dengue case.

All confirmed or epidemiologically linked cases of Dengue fever found in Health facilities from October 7 to 15, 2015 were included in the study. For the Controls, resident of Diredawa Town who was a neighbor to a case and who did not develop signs and symptoms of DF were included. On the other hand, Suspected Dengue fever patients who were critically ill and controls who were not a permanent resident of Diredawa city were excluded from the study. This study was carried out from October 7 to 15, 2015 at various private and public hospitals including health centers and private clinics of Diredawa. Health facilities were selected based on case load and all confirmed and epidemiologically linked cases at the health facilities during the investigation period were enrolled. Approval of the health facilities administration was obtained before approaching the patients. A total of 70 cases were interviewed with a ratio of 1:2 making the controls 140. Controls were neighbors of cases. After each case was interviewed at the hospital, their houses were visited and neighbors were interviewed.

The data was collected through face to face interview using structured questionnaire initially prepared in English and then translated to Amharic (Additional file 1). The questionnaire was divided in to three main areas covering demographic variables, risk factors associated with Dengue fever and Knowledge regarding the disease. For verifying consistency, a pre-tested questionnaire was used.

Sixty-nine serum samples were collected from malaria negative suspected patients by skilled professional and sent to Ethiopian Public Health Institute (EPHI) national laboratory. Samples were collected to identify the cause of the outbreak. The outbreak was confirmed first by RDT and then RT-PCR was performed for confirmation.

The data was checked for completeness and consistency and analyzed using SPSS version 20 software. Associations between factors and Dengue Fever status were tested first by the chi-square test and for cells with values less than 5, Fisher’s exact test was applied. In order to investigate relative importance of the variables in relation to the dependent factor and any confounding between them, they were fitted in a binary logistic regression model to identify independent factors. Those variables that come significant in the bivariate analysis were fitted to a multivariable analysis followed by a backward stepwise procedure to control confounding. Results were displayed using texts, tables and graphs and statistical significance was interpreted using Odd ratio with 95% confidence interval and *P* value < 0.05.

Ethical clearance was obtained from EPHI. A letter was written for the regional health bureau in order to obtain approval on the data collection. An informed consent was obtained from all study participants. Where the age was less than 16 years, assent was obtained from the children/adolescents and permission was obtained from respective parents/guardians. Confidentiality of information was assured and ensured. Participants were treated with respect and willingly participated in the study with no payment or coercion.

## Results

### Socio demographic characteristics

A total of 210 individuals (70 cases and 140 controls) were approached for interview. Response rate of the study was 100%, no decline of participant in the study individuals. From the total respondents, 94 were males and 116 were females. Out of the 70 cases, 34(48.6%) were males. The mean age for cases was 23.7 ± 9.5 SD and for controls 31.2 ± 13 SD. From the total cases, 39(55.7%) were singles and 31(44.3%) were married. Among the cases, more than half 39(55.7%) did not have occupation whereas, among the controls, those who work privately and those who do not have occupation were equal, 60(42.9%). Half of the cases 35(50%) have finished secondary school and only 16(23%) of them have managed to finish college or university. While for the controls 56(40%) have finished secondary school whereas, 20(14.3%) and 21(15%) were illiterate and reached college or university respectively (Table 1).

**Table 1:** Demographic characteristics of DF Cases and Controls, Diredawa city, October 2015

Characteristics	Cases *n* = 70	Controls *n* = 140	Total *n* = 210	*P*-Value	COR (95% CI)
Sex					
Male	34 (48.6%)	60 (42.9%)	94 (44.8%)		
Female	36 (51.4%)	80 (57.1%)	116 (55.2%)	0.433	1.259 (0.708–2.240)
Age group					
< 5	2 (2.9%)	0 (0%)	2 (0.95%)		
5–14	9 (12.9%)	7 (5.0%)	16 (7.6%)		
15–44	57 (81.4%)	113 (66.5%)	170 (81.0%)		
> 45	2 (2.9%)	20 (14.3%)	22 (10.4%)	**0.033**	5.044 (1.139–22.337)
Marital Status					
Single	39 (55.7%)	69 (49.3%)	108 (51.4%)		
Married	31 (44.3%)	71 (50.7%)	102 (48.6%)	0.380	1.295 (0.727–2.304)
Occupation					
No Occupation	39 (55.7%)	60 (42.9%)	99 (47.1%)		
Private Business	20 (28.6%)	60 (42.9%)	80 (38.1%)		
Government Employee	11 (15.7%)	20 (14.3%)	31 (14.8%)	0.272	0.606 (0.248–1.480)
Education					
Illiterate	6 (8.6%)	20 (14.3%)	26 (12.4%)		
Primary	13 (18.6%)	43 (30.7%)	56 (26.7%)		
Secondary	35 (50%)	56 (40%)	91 (43.3%)		
Tertiary	16 (22.9%)	21 (15%)	37 (17.6%)	0.617	0.820 (0.378–1.781)
Ethnicity					
Amhara	34 (48.6%)	50 (35.7%)	84 (40%)		
Oromo	22 (31.4%)	45 (32.1%)	67 (31.9%)		
Somali	8 (11.4%)	27 (19.3%)	35 (16.7%)		
Others	6 (8.6%)	18 (12.9)	24 (11.4%)	0.477	1.467 (0.511–4.213)

The numbers in Bold reflect significant variables with *P* value < 0.05

### Laboratory result

First 69 samples were analyzed by RDT at the field. Of which 26 were positive and the rest were negative. However, for further confirmation RT-PCR (Real time reverse transcription polymerase chain reaction) was done and 42 were confirmed positive, 20 were negative and 7 samples were not applicable (samples were inadequate). The rest of the cases were epidemiologically linked by person, place and time with the cases.

### Knowledge towards dengue fever

The interviewees were also asked knowledge questions regarding DF and out of the total cases, 34.2% of them heard about DF, while the rest 44.3% of the controls had a clue about the disease. Only 11.4% of the cases and 7.1% of the controls stated virus as the cause for DF. On the other hand, 27.1% of the cases and 26.4% of the controls knew the mode of transmission. Moreover, 90% of the cases and 91% of the controls had no idea regarding the time mosquito bites. Additionally, respondents were asked about the prevention of the disease and it was found that 75.7% of the cases and 73.6% of the controls were unaware of it. Even though more than half of the respondents didn’t hear about Dengue, almost 27% of the cases knew the symptoms (Table 2).

**Table 2:** Knowledge on Dengue Fever cases and controls, Diredawa city, 2015

Characteristics	Case *n* = 70	Control *n* = 140	Total *n* = 210	*P*-Value	COR (95% CI)
Heard About Dengue					
Yes	24 (34.2%)	62 (44.3%)	86 (40.0%)		
No	46 (65.7%)	78 (55.7%)	124 (59.01%)	0.166	1.524 (0.840–2.764)
Cause of Dengue					
Know	8 (11.4%)	10 (7.1%)	18 (8.6%)		
Don’t know	62 (88.6%)	130 (92.9%)	192 (91.4%)	0.300	1.677 (0.631–4.459)
Mode of transmission					
Know	19 (27.1%)	37 (26.4%)	56 (26.7%)		
Don’t Know	51 (72.9%)	103 (73.6%)	154 (73.3%)	0.912	1.037 (0.543–1.981)
Time Mosquito bites					
Know	7 (10%)	12 (8.6%)	19 (9.0%)		
Don’t Know	63 (90%)	128 (91.4%)	191 (90.9%)	0.734	1.185 (0.445–3.157)
Water required for breeding					
Know	17 (24.3%)	37 (26.4%)	54 (25.7%)		
Don’t Know	53 (75.7%)	103 (73.6%)	156 (74.2%)	0.738	0.893 (0.460–1.733)
Knew Symptoms					
Yes	19 (27.1%)	43 (30.7)	62 (29.5%)		
No	51 (72.9%)	97 (69.3%)	148 (70.5%)	0.593	0.840 (0.444–1.590)

### Risk factors towards dengue fever

From the total cases only 2.9% had previous exposure to the disease. The availability and use of Long-LastingInsecticidal Nets (LLINs) were assessed and it was found that 20% of the cases have LLINS while 37.9% of the controls had access to LLINs. Only 10% of the cases and 23.6% of the controls use it while sleeping. Though almost all respondents had water holding containers in their houses, there were no larvae identified in the households. Thirty-nine and 36 % of the cases stay at their home in the morning and afternoon respectively. While for controls, 33 and 37% are at home during the respective times. In addition to this, 18.6% of the cases were exposed to stagnant water around their house of which 80% of them living at a distance of < 100 m from the stagnant water. Moreover, none of the respondents’ house was sprayed in the last three months. In terms of close contact, 57% of the cases had close contact with a person of the same complaint, while only 19% of the controls had similar exposure. None of the cases use repellant on their skins while 2.9% of the controls use. Additionally, 17.1 and 15.7% of the cases and controls respectively use repellant in their houses. Moreover 39.5% of the total participants use window screen. Since Diredawa’s weather is warm, more than three quarter of the cases and controls (80%) each wear short sleeve clothes (Table 3).

**Table 3:** Risk factors towards Dengue Fever cases and controls, Diredawa city, October 2015

Characteristics	Cases *n* = 70	Controls *n* = 140	Total *n* = 210	*P*-Value	COR (95% CI)
Infected Previously					
Yes	2 (2.9%)	6 (4.3%)	8 (3.8%)		
No	68 (97.1%)	134 (95.7%)	202 (96.2%)	0.613	1.522 (0.299–7.744)
Availability of LLINs					
Yes	14 (20%)	53 (37.9%)	67 (32.0%)		
No	56 (80%)	87 (62.1%)	143 (68.1%)	**0.010**	2.437 (1.237–4.800)
Utilization of LLINs					
Yes	7 (10%)	33 (23.6%)	40 (19.0%)		
No	63 (90%)	107 (76.4%)	170 (81.0%)	**0.022**	2.776 (1.159–6.645)
Water Holding Container					
Yes	56 (80%)	131 (93.6%)	187 (89.0%)		
No	14 (20%)	9 (6.4%)	23 (11.0%)	**0.005**	3.639 (1.488–8.896)
Stagnant water in the village					
Yes	13 (18.6%)	9 (6.4%)	22 (10.5%)		
No	57 (81.4%)	131 (93.6%)	188 (89.5%)	**0.009**	0.301 (0.122–0.745)
close contact in the last 2 weeks					
Yes	40 (57.1%)	27 (19.3%)	67 (31.9%)		
No	30 (42.9%)	113 (80.7%)	143 (68.1%)	**0.000**	0.179 (0.095–0.337)
Remained at home in the morning from 8 to 10 am					
Yes	27 (38.6%)	46 (32.9%)	73 (34.8%)		
No	43 (61.4%)	94 (67.1%)	137 (65.2%)	0.413	0.779 (0.429–1.415)
Remained at home in the afternoon 4–6 pm					
Yes	25 (35.7%)	49 (35%)	74 (35.2%)		
No	45 (64.3%)	91 (65%)	136 (64.8%)	0.919	0.969 (0.532–1.766)
Use of mosquito repellant in the House					
Yes	12 (17.1%)	22 (15.7%)	34 (16.2%)		
No	58 (82.9%)	118 (84.3%)	176 (83.8%)	0.791	0.901 (0.417–1.947)
Use of mosquito repellant in the skin					
Yes	0 (0%)	4 (2.9%)	4 (1.9%)		
No	70 (100%)	136 (97.1%)	206 (98.1%)	0.304	
Type of cloths					
Short sleeves	56 (80%)	112 (80%)	168 (80%)		
Long Sleeves	14 (20%)	28 (20%)	42 (20%)	1.000	1.000 (0.488–2.049)

Availability and utilization of LLINs (*p* = 0.010 and 0.022 respectively), availability of water holding container (*p* = 0.005), close contact with DF patient (*p* = 0.000) and the availability of stagnant water around the house (*p* = 0.009) were important risk factors (Table 3).

After backward logistic regression was performed, three variables remained significant: utilization of LLINs (adjusted OR = 2.74: 95% CI: 1.06–7.08), availability of stagnant water (adjusted OR = 3.61: 95% CI: 1.31–9.93) and close contact with DF patients (adjusted OR = 5.36:95% CI: 2.75–10.44) (Table 4).

**Table 4:** Independent predictors of Dengue Fever, Dire Dawa, October 2015

Characteristics	*P*-Value	S. E	AOR (95%CI)
Close contact			
Yes			5.36 (2.75–10.44)
No	0.000	0.340	1.0
Utilization of LLINs while sleeping			
Yes			1.0
No	0.034	0.486	2.74 (1.06–7.08)
Stagnant water in the village			
Yes			3.61 (1.31–9.93)
No	0.017	0.509	1.0

## Discussion

The study showed that the risk factors of DF in Diredawa administration city were the availability of stagnant water around the house, the non-use of LLINs and having close contact with Dengue Fever patient. Those who have stagnant water around their houses were 3.6 times more at risk to have the disease. In consistence to this, results of a case control study carried out in Vietnam identified that people living near stagnant water like ponds, lakes or open sewers had higher rates of morbidity [12]. The DF epidemic in Brazil was also associated with proximity to uncontrolled water ways and stagnant water in tanks, gutters and cans [13]. This may be due to the fact that, *Aedes aegypti* breeds on artificial and natural water containers almost in and around households, construction sites, factories, etc. [14].

The current study has also found a significant association between Dengue fever and close contact with DF patient. Having close contact with DF patient has a 5.4 times risk in acquiring the disease. This may be because of the likely availability of *Aedes aegypti* breeding site around the sick person’s living environment which will also be a risk factor for a person spending more time with the patient.

Additionally, there is an association found between the uses of LLINs and DF showing that people who do not use LLINs were 2.7 times more likely to get infected than those who use. Another study done in Angola has also found that Behavior associated with protection from DENV infection included having recently used mosquito avoidance strategies (such as applying mosquito repellent or sleeping under a bed net) were protective from the disease [15]. On the contrary, study in Zambia revealed that up to 21% of the respondents using insect trapping nets (ITNs) were more likely to be infected with Dengue fever. This may be attributed to the fact that the mosquito *Aedes aegypti* commonly bites during the day and the use of ITNs while sleeping at nights would not be expected to provide a barrier between the humans and the vector unless it is used while sleeping during the day time [16].

The knowledge of the community regarding DF was also assessed and it was found that only 41% of the participants have heard about DF previously out of which 34% were cases. However, in a study conducted in Karachi, 96.5% of high socio-economic population and 88% of low socio-economic population have heard about the disease [17]. This discrepancy may be due to the recurrent occurrence of DF in Pakistan. Regarding the breeding site of the mosquito, only about a quarter of the cases had idea that water is required for the mosquito to breed. Whereas the study in Lahore [18] had showed that almost half of the patients knew stagnant water as a favorable breeding site. This difference may be due to the dengue awareness campaigns given in Lahore after the 2011 outbreak. Only 27% of the cases could identify the vector as a mosquito, while in Lahore 72% of Dengue positive patients were aware that mosquito transmits the disease. Similarly, 97.3% of the participants in the study in Vietnam identified *Aedes aegypti* as the vector [19]. Regarding symptoms, 27% of the patients knew the symptoms of DF, and this finding contradicts with the Lahore study, where 88% of the cases knew DF symptoms. The low awareness of the people about the disease may be due to the fact that the disease is not common in our country and mostly undetected.

Even though there was full response rate in this study, we interviewed only patients that appeared to health facilities and so may have missed factors related to cases that did not seek medical care. Moreover, controls were not willing to give blood samples and therefore it was not possible to confirm whether they were really free of the disease.

## Conclusions

Presence of stagnant water around the house, not using LLINs while sleeping and having close contact with DF patient were independent risk factors for disease contraction.

In the absence of vaccination and effective drugs, the only intervention is vector control to contain the outbreak and prevent future occurrences. Therefore, draining of stagnant water, giving health education in LLINs use and early medical seeking for persons living with DF patients is needed. The vector control activity needs to be part of long-term control of DF that will also contribute to controlling other diseases with the same determinants.

## Additional file

Additional file 1: Questionnaire - Dengue Fever outbreak. Factors associated with Dengue Fever Outbreak-Dire Dawa administration city, October 2015. (DOC 173 kb)

## Data Availability

The datasets used and/or analyzed during the current study are available from the corresponding author on reasonable request.

## Abbreviations

AFENET: African field epidemiology network
AOR: Adjusted odds ratio
CDC: Center for disease control and prevention
CFR: Case fatality rate
COR: Crude odds ratio
DF: Dengue fever
DHF: Dengue hemorrhagic fever
DSS: Dengue shock syndrome
ELISA: Enzyme linked immune sorbent assay
EPHI: Ethiopian Public Health Institute
IRS: Indoor residual spray
LLINs: Long Lasting Insecticidal Nets
OPDs: Outpatient Departments
RDT: Rapid diagnostic test
RT-PCR: Real time polymerase chain reaction
SPSS: Statistical package for social sciences
WHO: World Health Organization
