# Supplementary material for: Factors associated with dengue fever outbreak in Dire Dawa administration city, October, 2015, Ethiopia - case control study
Source: BMC Public Health. 2019 May 28;19:650. doi: 10.1186/s12889-019-7015-7 (PMC6540423; doi:10.1186/s12889-019-7015-7)
Supplement: Supplementary file 1 — Questionnaire - Dengue Fever outbreak. Factors associated with Dengue Fever Outbreak-Dire Dawa administration city, October 2015. (DOC 173 kb) [file 12889_2019_7015_MOESM1_ESM.doc]

# Consent Form:

Hello, my name is______________________. I am here on be behalf of the researcher.

We would like to understand the factors associated with Dengue Fever Outbreak in Diredawa administration city. To get this information, we are caring out interviews, now I will ask you to complete this questionnaire about socio-demography, knowledge and exposure towards Dengue Fever. The results of this research will help to identify the factors associated with Dengue Fever and control the outbreak. The interview takes about 30 minutes. What you tell me will be kept strictly confidential and no one other than the researcher will find out the answers that you give. Your name and address or child’s status will never appear on this study separately. Participation is voluntary and you may withdraw from the session at any time or refuse to answer any questions that makes you uncomfortable. Participation in this study will not affect your personal dignity. If you have further questions about the study, you can contact Luna Habtamu Mob. +251-913-77-55-92 or e-mail lunahatamu403@gmail.com.

Are you willing to take part in the interview? Yes No

Thank you for your participation. We are very grateful for your help.

Informed consent certified by:

Interviewer name_______________________ signature___________________

Date of interview__________________________

Result of interview: 1. Completed 2. Completed partially 3. Refused

**Factors associated with Dengue Fever Outbreak-Dire Dawa administration city, October 2015**

| **No.** | **Question** | | | **Coding Classification** | | **Go To** |
| --- | --- | --- | --- | --- | --- | --- |
| 1. **Demographic Information Kebele _________ House No_________** | | | | | | |
|  | Respondent ID | | | _____________________ | |  |
|  | Sex | | | 1.Male  2.Female | |  |
|  | Age | | | _______Years | |  |
|  | Marital Status | | | 1. Single  2. Married  3. Divorced  4. Widowed  5.NA | |  |
|  | Educational status | | | 1. Illiterate  2.Primary school  3. Secondary  4. College And University | |  |
|  | Ethnicity | | | 1. Somali  2. Amhara  3. Oromo  4. Tigre  5. Other(specify) | |  |
|  | Occupation | | | 1. Student  2. Daily laborer  3. House wife  4. Merchant  5.Farmer  6.Other(specify) | |  |
|  | Number of family members? | | | __________ | |  |
| 1. **Clinical Information** | | | | | | |
|  | Respondent Classification | | | 1. Cases  2. Controls | | If Control skip to 4.1 |
|  | Date of Onset | | | dd/mm/yyyy ___________ | |  |
|  | Sign and symptoms | | | 1.Fever  2. Headache  3. Chill  5. Nasal Bleeding/bleeding from any part of the body  6. severe muscle and [joint pain](http://www.medicinenet.com/script/main/art.asp?articlekey=24725)  7. Rash  8. Restlessness/lethargy  9. Abdominal pain, Nausea/vomiting  10.Other(specify) | |  |
|  | Date seen at health Facility | | | dd/mm/yyyy ___________ | |  |
|  | Treatment | | | Antibiotics ____________  Antiviral ______________  Antipyretics____________  Ant malaria___________  Other supportive treatment | |  |
| 1. **Laboratory Specimens** | | | | | | |
|  | Is sample taken for dengue Fever? | | | 1. Yes  2. No | |  |
|  | Date sample collected? | | | _____/______/______ | |  |
| 1. **Knowledge towards Dengue fever** | | | | | | |
|  | Do you hear about Dengue fever? | | | 1.Yes  2.No | |  |
|  | If yes, what do you think the cause of Dengue fever? | | | 1.Virus  2.Bacteria  3.protozoa  4.other____  5. Don’t know | |  |
|  | Is Dengue fever Contagious? | | | 1.yes  2.No  3. I Don’t know | |  |
|  | If yes, how is it transmitted? | | | 1.By mosquito  2. Air droplets  3.House fly  4.Other___  5. Don’t know | |  |
|  | At which time mosquito bites people? | | | 1. Night  2. Day  3.Sunrise/sunset  4. I Don’t know | |  |
|  | Does water required for mosquito to breed? | | | 1.Yes  2.No  3. I Don’t know | |  |
|  | Do you know symptoms of dengue fever? | | | 1.Yes  2.No | |  |
|  | If yes what are the symptoms? | | | 1.Fever  2. Headache  3. bleeding  4. Rash  5. Other(specify)_____ | |  |
| 1. **Exposure** | | | | | | |
|  | Have you ever been infected by Dengue fever? | | | 1.Yes  2.No | |  |
|  | If Yes, when? | | | _________ Year | |  |
|  | Do you have LLINs? | | | 1.Yes  2.No | |  |
|  | If Yes, do you use LLINs while sleeping? | | | 1.Yes  2.No | |  |
|  | When did you get the last LLINs? | | | _______ Year | |  |
|  | Who use LLINs always? | | | 1.Children  2.Women  3. All use equally | |  |
|  | Is there any water holding container in/around the house? | | | 1. Yes  2. No | |  |
|  | If yes, what is the type of the container? Multiple answer possible | | False Banana Used pots  Jerry can  Garbage disposal that contain water  Flower pots/vases | | Used tyres  Discarded food and beverage containers  Blocked gutters  Buildings under construction  Others (Specify)___________ | |
|  | Is there Aedes Mosquito larvae identified in the water containers in/around the house? Observe | | | | 1.Yes  2.No | |
|  | If yes, which water container has larvae in side? Tick all possible | False Banana Used pots  Jerry can  Garbage disposal that contain water  Flower pots/vases | | | Used tyres  Discarded food and beverage containers  Blocked gutters  Buildings under construction  Others (Specify)___________ | |
|  | Are you usually around your home in the morning from 8-10am? | | | | | |
|  | Are you usually around your home in the afternoon from 4-6pm? | | | | | |
|  | Is there any stagnant water around your village? | | | 1.Yes  2.No | |  |
|  | If Yes, approximately distance of stagnant water from your house? | | | 1.less than 100m  2.more than 100m | |  |
|  | Is your house sprayed? | | | 1.Yes  2.No | |  |
|  | When was the last time that your house sprayed? | | | 1.One month ago  2.Two months ago  3, Three months ago  4. More than three months | |  |
|  | Is there any river around your village? | | | 1.Yes  2.No | |  |
|  | If Yes what is the name of the river? | | | ___________ | |  |
|  | Approximately distance of the river from your house? | | | 1.less than 100m  2.more than 100m | |  |
|  | Is there any person diseased in your family? | | | 1.Yes  2.No | |  |
|  | If Yes, how many family members become ill? | | | _________ | |  |
|  | Did you have close contact with person with same complaint within the last 1 to 2 weeks? | | | 1.Yes  2.No | |  |
|  | Did you have travel history within the last two weeks? | | | 1. Yes 2. No | |  |
|  | If, yes to where? | | | _______ | |  |
|  | Do you use air conditioning or window and door screens? | | | 1.Yes  2.No | |  |
|  | Do you use mosquito repellents on your skin? | | | 1.Yes  2.No | |  |
|  | Do you use mosquito repellant in your house? | | | 1. Yes 2. No | |  |
|  | What kind of clothes you usually wear | | | 1. Short and T-shirts  2. Trousers/ body full dress | |  |

***Thank you for your cooperation***
